# Distribution of 45S rDNA sites in chromosomes of plants: Structural and evolutionary implications

**DOI:** 10.1186/1471-2148-12-225

**Published:** 2012-11-26

**Authors:** Fernando Roa, Marcelo Guerra

**Affiliations:** 1 Department of Botany Laboratory of Plant Cytogenetics and Evolution, Federal University of Pernambuco Center of Biological Sciences, Rua Nelson Chaves, s/n Cidade Universitária, Recife, PE, 50.670-420, Brazil

**Keywords:** 45S rDNA sites, Chromosome regions, Secondary constrictions, NOR, Meta-analysis

## Abstract

**Background:**

45S rDNA sites are the most widely documented chromosomal regions in eukaryotes. The analysis of the distribution of these sites along the chromosome in several genera has suggested some bias in their distribution. In order to evaluate if these loci are in fact non-randomly distributed and what is the influence of some chromosomal and karyotypic features on the distribution of these sites, a database was built with the position and number of 45S rDNA sites obtained by FISH together with other karyotypic data from 846 plant species.

**Results:**

In angiosperms the most frequent numbers of sites per diploid karyotype were two and four, suggesting that in spite of the wide dispersion capacity of these sequences the number of rDNA sites tends to be restricted. The sites showed a preferential distribution on the short arms, mainly in the terminal regions. Curiously, these sites were frequently found on the short arms of acrocentric chromosomes where they usually occupy the whole arm. The trend to occupy the terminal region is especially evident in holokinetic chromosomes, where all of them were terminally located. In polyploids there is a trend towards reduction in the number of sites per monoploid complement. In gymnosperms, however, the distribution of rDNA sites varied strongly among the sampled families.

**Conclusions:**

The location of 45S rDNA sites do not vary randomly, occurring preferentially on the short arm and in the terminal region of chromosomes in angiosperms. The meaning of this preferential location is not known, but some hypotheses are considered and the observed trends are discussed.

## Background

When observed under an optical or electronic microscope, the chromosomes of plants and other eukaryotes seem to be uniform structures, except for the primary and secondary constrictions. However, based on some functional and molecular characteristics, at least five distinct regions can be recognized along each chromosome arm: telomere, subtelomeric region, interstitial region, proximal region and centromere. The distribution of genes, retrotransposons, satellite DNA and other sequences within these regions seems to follow general trends related to the frequency of recombination and the functional role of these sequences [1-4]. Therefore, the structural regularity does not reflect the different selection pressures that different regions of the chromosome arm suffer, creating sub-regions more permissive or more prohibitive for the establishment of a specific sequence [4-6].

Lima-de-Faria [7] showed that the secondary constrictions of mitotic chromosomes, which bear the 18S-5.8S-25S ribosomal RNA genes transcribed in the previous interphase, also called the nucleolus organizer regions (NORs) or 45S rDNA sites, were preferentially distributed on the short arms and in the subterminal region in most species of plants and animals. The author compared the frequency of secondary constrictions in the proximal, interstitial and terminal regions of the chromosome arm, taking into account the size of the arms (*arm frame method*). Currently, by using the fluorescence *in situ* hybridization (FISH) technique, it is known that the secondary constrictions represent only the expression of rRNA genes which were active during the last interphase and that other functional sites may not form secondary constrictions, especially if located too close to the terminal end of the chromosomes [8]. In *Vigna unguiculata*, for example, three pairs of rDNA sites not previously detected as secondary constrictions were revealed by FISH [9]. The fact that the 18S-5.8S-25S rRNA genes are arranged in hundreds or thousands of tandem repeats and are highly conserved between species made of this chromosome region the most widely investigated by FISH, being the position of these sites known in hundreds of plant species, especially in angiosperms.

In this study, the general pattern of distribution of rDNA sites in plant chromosomes was addressed to establish whether these genes are randomly distributed or occupy preferred regions on chromosome and whether certain chromosomal or karyotypic characteristics influence this distribution. Karyotypic data including information about rDNA sites of 846 species of angiosperms and gymnosperms were compiled from the literature. Other groups of plants have few representatives studied and do not allow generalizations. In pteridophytes, the rDNA sites have been observed only in four genera (*Osmunda*, *Ceratopteris*, *Acrostichum*[10] and *Selaginella*[11]), all of them with terminal or subterminal sites. In bryophytes, data were found only for *Marchantia polymorpha*[12] and species of *Pellia*[13], all of them with rDNA sites in terminal position. In a recent paper, a rDNA database containing crude data from a similar sample of plant species has been developed allowing easy access to a large amount of data [14].

In some cases, the analysis was done separately in karyotypes containing only one pair of sites and karyotypes with more than one pair of sites, considering that in those with only one pair of sites they should always be functionally active. In karyotypes with multiple sites the assumption that they had a tendency to equilocal distribution [15-17] was evaluated. As polyploid plants tend to diploidize reducing the original monoploid genome size [18,19], the effect of polyploidy in the number and position of rDNA sites in the intraspecific and intrageneric levels was also evaluated.

## Results and discussion

### Number of rDNA sites in angiosperms and gymnosperms

In this paper we compiled all the information about number and position of rDNA sites we had access until early 2011. A total of 846 species and 198 genera were included, representing 51 families of angiosperms and six families of gymnosperms. Among angiosperms, 42 species stand out for having holokinetic or holocentric chromosomes, i.e., chromosomes with a large kinetochore plate extended along almost their whole length [20], which will be treated in a separate topic. Since some species were polymorphic or had different cytotypes, a total sample of 984 karyotypes was analyzed, 921 and 63 of them having monocentric or holokinetic chromosomes, respectively.

**Figure 1:**
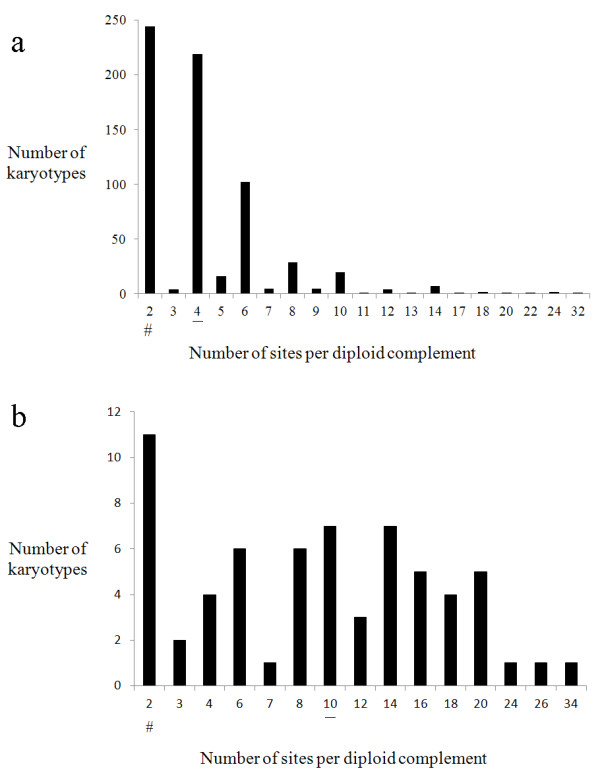
**Variation in the number of rDNA sites per karyotype in a sample of 665 angiosperm (a) and 64 gymnosperm (b) karyotypes.** Polyploid cytotypes and species with holokinetic chromosomes were not included. The median is underlined and the modal number is indicated by #. Sample size: 729 karyotypes.

The results obtained here seem to represent well the diversity in plants, because of the wide diversity of taxa and karyotypic variations sampled. The number of rDNA sites observed was very variable, ranging from just one pair of sites per chromosome complement in most angiosperms up to 45 sites in *Kyllinga brevifolia*[21]. In angiosperms with monocentric chromosomes, the average number of sites per somatic chromosome complement was 5.1 (3.6) (mean and standard deviation) ranging from two to 32, with mode equal to two and a median of four (Table 1). Figure 1a shows that there was a high frequency of diploid karyotypes with two or four sites and a progressive reduction in the number of karyotypes with higher numbers of sites. When plolyploids were included in the sample the modal number of sites per karyotype (2) differed from the second more common number (4) by a very small difference: 265 out of the 856 karyotypes had two sites per somatic chromosome complement, whereas 263 karyotypes had four and 328 had three or more rDNA sites. The modal number of rDNA sites in angiosperms can easily oscillate between two and four depending on the sampling.

**Table 1:** Number of rDNA sites per somatic chromosome complement (2n) in species of angiosperms and gymnosperms

	**Angiosperms**		**Gymnosperms**	**Total**
	**Monocentrics**	**Holokinetics**		
Number of karyotypes	856	63	65	984
Mean of sites (s.d.)	5.1 (3.6)	6.6 (6.6)	10.4 (7.1)	5.5 (4.4)
Median	4	4	10	4
Mode	2	4	2	4
Range	2 - 32	2 - 45	2 - 34	2 – 45
Karyotypes with single site*	265 (31.0%)	6 (9.5%)	11 (16.9%)	282 (28.7%)

* Karyotypes with a single pair of sites.

In gymnosperms, the average number of sites 10.4 (7.1) was more than twice that in angiosperms, although the modal number of sites was two, as in angiosperms (Table 1, Figure 1b). The presence of multiple sites in some genera as *Picea*[22], *Pinus*[23,24] and *Zamia*[25], upraised the median of this group to 10. In general, gymnosperms have a larger number of copies of these genes [26] and a larger average genomic size than angiosperms, probably meaning that they are more tolerant to amplifications [27]. However, a larger number of 45S rDNA sites does not mean more functional copies of these genes, either because the size of the sites is very variable or because the rDNA probe can also detect non-functional or permanently blocked copies of these genes [28,29]. In karyotypes with a single pair of 45S rDNA sites, here referred as single site karyotypes, both sites are necessarily functional whereas karyotypes with multiple sites may eventually include some non-functional or inactive sites. Therefore, in some cases it is important to visualize the distribution of single site and multiple sites karytotypes separately.

rDNA sites often display intraspecific heteromorphism in number and size (see e.g. [30,31]). For example, in the common bean *Phaseolus vulgaris*, we found that the number of 45S rDNA sites varied from six to 18 per accession and their size varied widely within chromosome pairs [32]. Some sites may be below the limit of detection by FISH and their eventual amplification in different populations may account for the numerical polymorphism. It is also possible that inactive rDNA sites are more likely to show intraspecific polymorphisms and eventually be eliminated [33]. This dynamics of inactivation and subsequent deletion of rDNA sites seem to counteract the mechanisms of duplication and dispersion of 45S rDNA repeats, leading to the observation of only a small number of sites in most of the species.

### Distribution of rDNA sites along the chromosome

**Figure 2:**
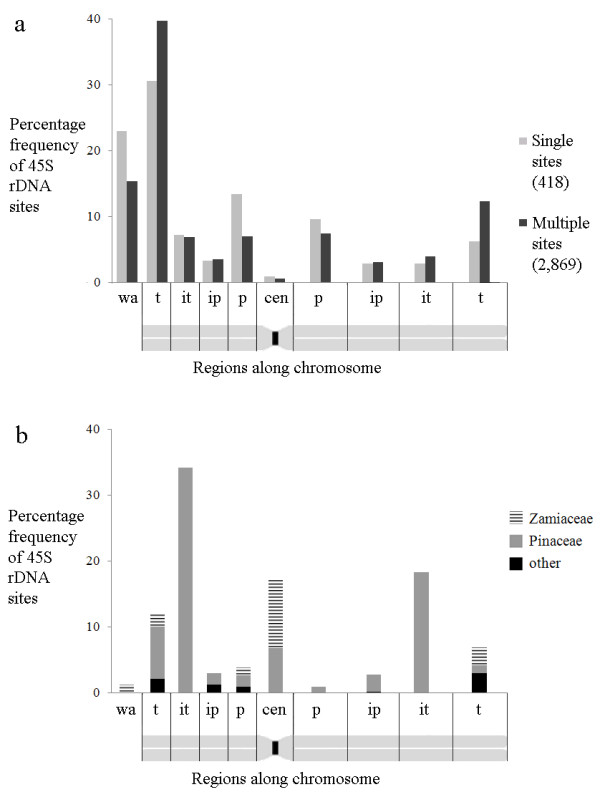
**Percentage frequency of rDNA sites along chromosomes of angiosperms (a) and gymnosperms (b).** Total number of sites: 3.287 in a and 679 in b. In **b**, the only eleven karyotypes with single sites, were represented together with multiples sites. To stress that some distributions are particular for some gymnosperm families, they were shown distinctly (cen, centromeric region; p, proximal; ip, interstitial-proximal; it, interstitial-terminal; t, terminal; wa, whole arm sites).

729 karyotypes of angiosperms and gymnosperms have been used for determining the number of rDNA sites per chromosomal arm, 509 of them displaying multiple sites (Table 2, Figure 2). The total number of rDNA sites included in this sample was 3,966. In some karyotypes the position of the site was just between the two chromosome arms and the signal seemed to coincide exactly with the position of the centromere. In these cases, the sites were referred to as "centromeric" (cen), without implying that they were really located in the functional region of the centromere. In general, 69.9% of the rDNA sites were located on the short arm. The frequency of rDNA sites in the short arm was higher among karyotypes with single sites (77.7%) than in those with multiple sites (68.9%), suggesting that when the number of sites increases, the trend to be preferentially positioned on the short arm decreases. According to Lima-de-Faria [7] 86.6% of secondary constrictions of animal and plant species occur on the short arms, a value higher than that observed here (p <0.01). This divergence is at least partially due to the techniques employed. In the case of Lima-de-Faria, he detected only secondary constrictions, that is, rDNA sites which were active in the last interphase, whereas in the present report, using FISH, all kind of 45S rDNA cluster, including non-active and very small ones, were detected.

**Table 2:** Frequency of rDNA sites in the short and long chromosome arms

**Sites**	**Number of karyotypes**	**Short arms**	**Centromeric sites**^**a**^	**Long arms**	**Total**	**p-value**^**b**^
Single^c^	220	342 (77.7%)	4 (0.9%)	90 (20.9%)	440	0.000001
Multiple	509	2,430 (68.9%)	132 (3.7%)	964 (27.3%)	3,526	0.000001
Total	729	2,772 (69.9%)	138 (3.5%)	1,056 (26.6%)	3,966	0.000001

^a^ Sites between the short and long arms whose exact position was not identified.

^b^ Chi-square comparison against an equal distribution of sites per arm.

^c^ Karyotypes with a single pair of sites.

In angiosperms, we observed that rDNA sites occupied preferentially (50.1%) the terminal position of the chromosomes, both in karyotypes with single site (36.8%) and in karyotypes with multiple sites (52.0%) (Figure 2 and Additional file 1: Table S1). It is worth mentioning that 23.0% of the single sites and 15.5% of multiple sites were represented by signals that occupied the whole chromosome arm (wa sites) and these sites were not computed as terminal ones. In many karyotypes, whole arm sites should represent a technical artifact generated by over-exposition of the signal during image acquirement whereas in other karyotypes they seem to correspond to the whole visible chromatin of the short arm, as in species of *Nothoscordum* and *Ipheion*[34]. If they were included as terminal sites, the frequency of rDNA sites in the terminal region would be more similar for single (59.8%) and multiples sites (67.5%). A higher percentage of terminal secondary constrictions (85.2%) was observed by Lima-de-Faria in a sample of 189 species of eukaryotes (p<0.01) including two families of plants [35]. One of them (Asteraceae) showed also a higher percentage of terminal rDNA sites in the present study. Whole arm sites were always located on the short arm (Figure 2), except in *Solanum pennellii*, where it spanned the entire slightly longer arm [36]. Therefore, considering that the chromosome arm was divided into four regions of equal size (p, ip, it, t), there seems to be a strong positive selection favoring the location of 45S rDNA sites in the terminal region. On the other hand, in gymnosperms the rDNA sites were located mainly in the interstitial-terminal (52.4%) or proximal (21.8%) regions. These frequencies were strongly influenced by members of Pinaceae and Zamiaceae families which had a large number of species investigated, each one displaying multiple sites. The terminal position of rDNA sites (18.9%) was largely dominant in the less sampled families of gymnosperms.

The location of rDNA sites in the terminal chromosome region in most angiosperms may be the result of homologous recombination constrains. Due to its repetitive organization in tandem, rDNA sites may be subject to a higher rate of allelic and non-allelic homologous recombinations, which play a fundamental role in the homogenization of intralocus and interloci repeats [37]. Homogenization is an essential process to reduce the nuclear variability of this key molecule, ensuring the removal of non-functional units from the clusters [38]. The terminal or subterminal position of rDNA sites allows the occurrence of rearrangements without deleterious effects related to gene balance and meiotic segregation [32,39]. Nonetheless, various genera of plants and animals have interstitial or proximal 45S rDNA sites [40] and display at least partial inter-loci homogenization [41,42]. In addition, repetitive sequences of 45S rDNA can also protect the telomere, in a way similar to subtelomeric DNA [43-45]. In *Allium cepa*, 45S rDNA acts as a substitute of telomeric DNA at some chromosomal termini [43].

### The particular case of acrocentric chromosomes

During the compilation of the data, it was often observed that acrocentric chromosomes had rDNA sites on the short arm, as for example in species of *Ipheion*[35], *Nothoscordum*[34,46], *Rumex*[47], and *Alstroemeria*[48]. To assess whether the short arm of acrocentric chromosomes is a especially preferred region for rDNA cluster location, 266 species with single site or multiple sites having at least one acrocentric chromosome per karyotype were analyzed. In this sample, 36.2% of the chromosomes were acrocentrics and 53.6% of the sites occurred on the long or short arm of these chromosomes. Considering only the acrocentric chromosomes, 64.7% of the sites occurred on the short arm and most of them (70.1%) occupied the entire short arm (wa sites). Therefore, considering that the short arm corresponds to no more than 25% of the length of the acrocentric chromosome [49], there is indeed a very high frequency of rDNA sites in the short arms of acrocentrics.

The rationale for this association is not clear. In some karyotypes the occurrence of a whole-arm site seems to be associated with a centric fission event, as observed in a rearranged karyotype of *Hypochaeris radicata*[50] and in species of *Ipheion*[35] and *Nothoscordum*[34,51]. Strong evidence for the association of rDNA with fission was also reported for hymenoptera and mice [42,52]. In these cases, the centric fission may have been preceded by insertion of 45S rDNA repeats in the centromeric region of a bi-armed chromosome, or both telocentrics may have acquired rDNA sequences in the new terminal regions after fission [50]. However, not all karyotypes with centric fission have extra rDNA sites and in some cases they may have the number of rDNA sites reduced [53]. In addition, though 45S rDNA are found on the short arms of acro/telocentric chromosomes in several plant and animal species, usually they are not associated with centric fissions [40]. Alternatively, the very short arms of acrocentric chromosomes may be a more accessible site for rDNA transposition and may allow a quicker interloci homogenization of rDNA sequences than the proximal region of metacentric chromosomes [54].

### Distribution of rDNA sites in different taxa

**Figure 3:**
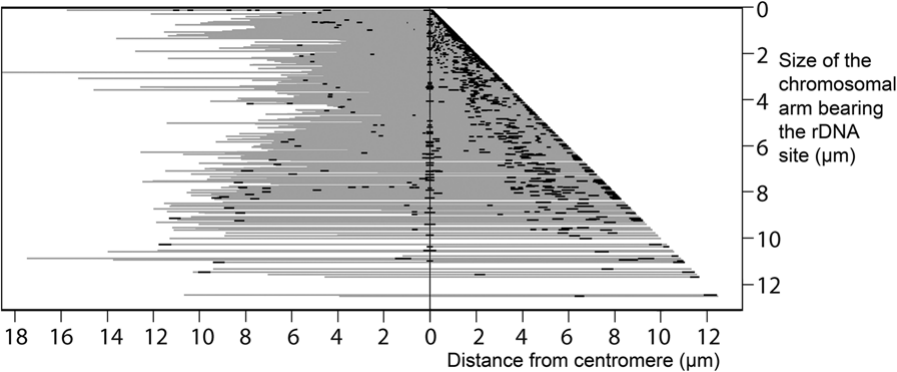
**Distribution of 45S rDNA sites in the arm frame.** Chromosomes are represented as grey lines aligned by centromeres (0) and sites as short bold lines. 46 chromosomes with sites in both arms were drawn twice in opposite directions. Sample size: 419 angiosperms and 38 gymnosperms karyotypes (383 and 37 species, respectively).

**Figure 4:**
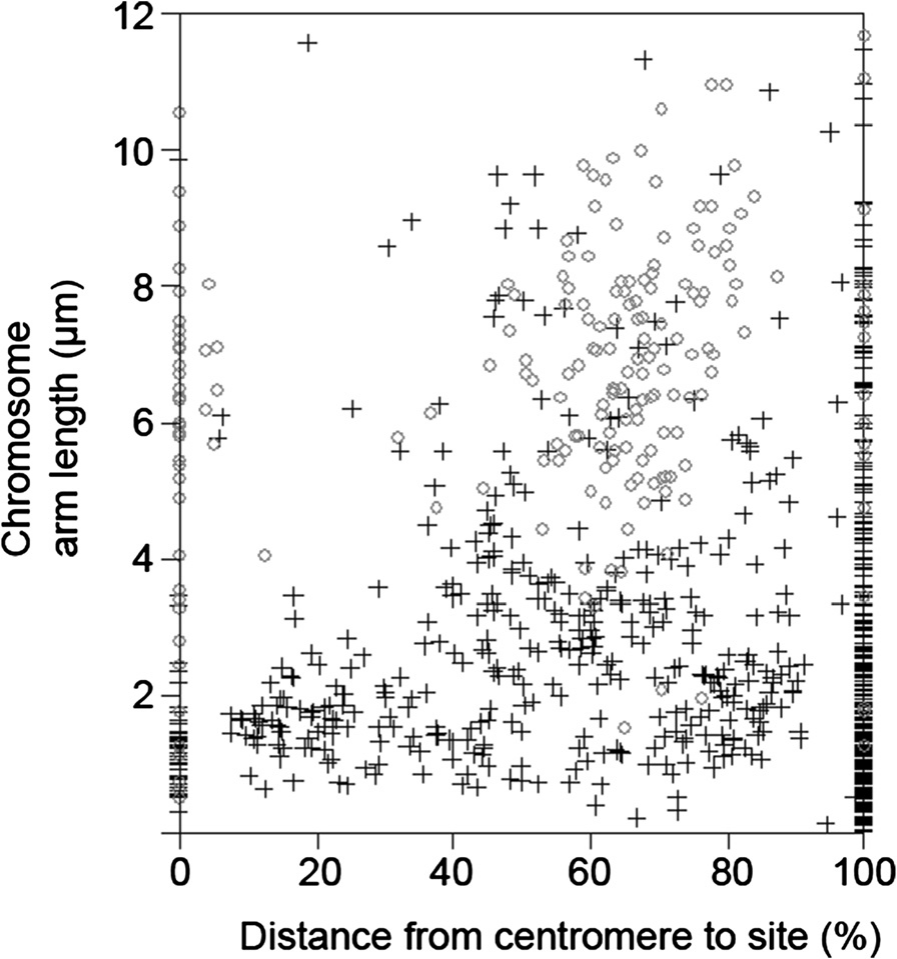
**Relative distribution of rDNA sites along the chromosome arm in 397 angiosperm (+) and 38 gymnosperm (o) karyotypes.** The distance from the site to the centromere was represented as a percentage value in relation to the centromere-telomere length of the arm bearing the rDNA site. Whole arm (wa) sites were not included in this sample.

An analysis of the distribution of rDNA sites by the arm frame method was carried out both in the whole sample and separately by family, aiming to identify specific trends (details of the arm frame construction are indicated in Material and Methods. 1201 rDNA bearing chromosomes from 457 karyotypes (420 species) with single site or multiple sites were included in this analysis. The graph of the arm frame revealed a higher concentration of sites in the terminal region while the proximal half of arms was particularly poor in sites (Figure 3), as described for the distribution of secondary constrictions in chromosomal arms [7]. Because the resolution of sites in the smaller arms was not very clear, the data of absolute distances of the sites to the centromeres were converted to relative distances (considering the centromere-telomere distance as 100% of the arm). Since angiosperms and gymnosperms have different distribution patterns of rDNA sites (Figures 1 and 2), these two groups of plants were distinctly represented in Figure 4. In this figure we can see that the higher occurrence of interstitial sites may be associated to gymnosperms, as previously stated, but it is also influenced by the different chromosome arm sizes. The interstitial distribution of sites on chromosome arms larger than 2 or 3 μm is characteristic of the largely sampled Poaceae [55,56] while in shorter arms the site position is more variable (Figure 4, Additional file 2: Figures S1, S2). The statement of Lima-de-Faria [7] that as the distance between centromere and telomere increases the ribosomal genes are successively displaced maintaining a relative distance to centromere and telomere seems to apply for most genera but not for every family. This is especially apparent when the position of sites is visualized in terms of percentages (Additional file 2: Figures S1, S2).

**Figure 5:**
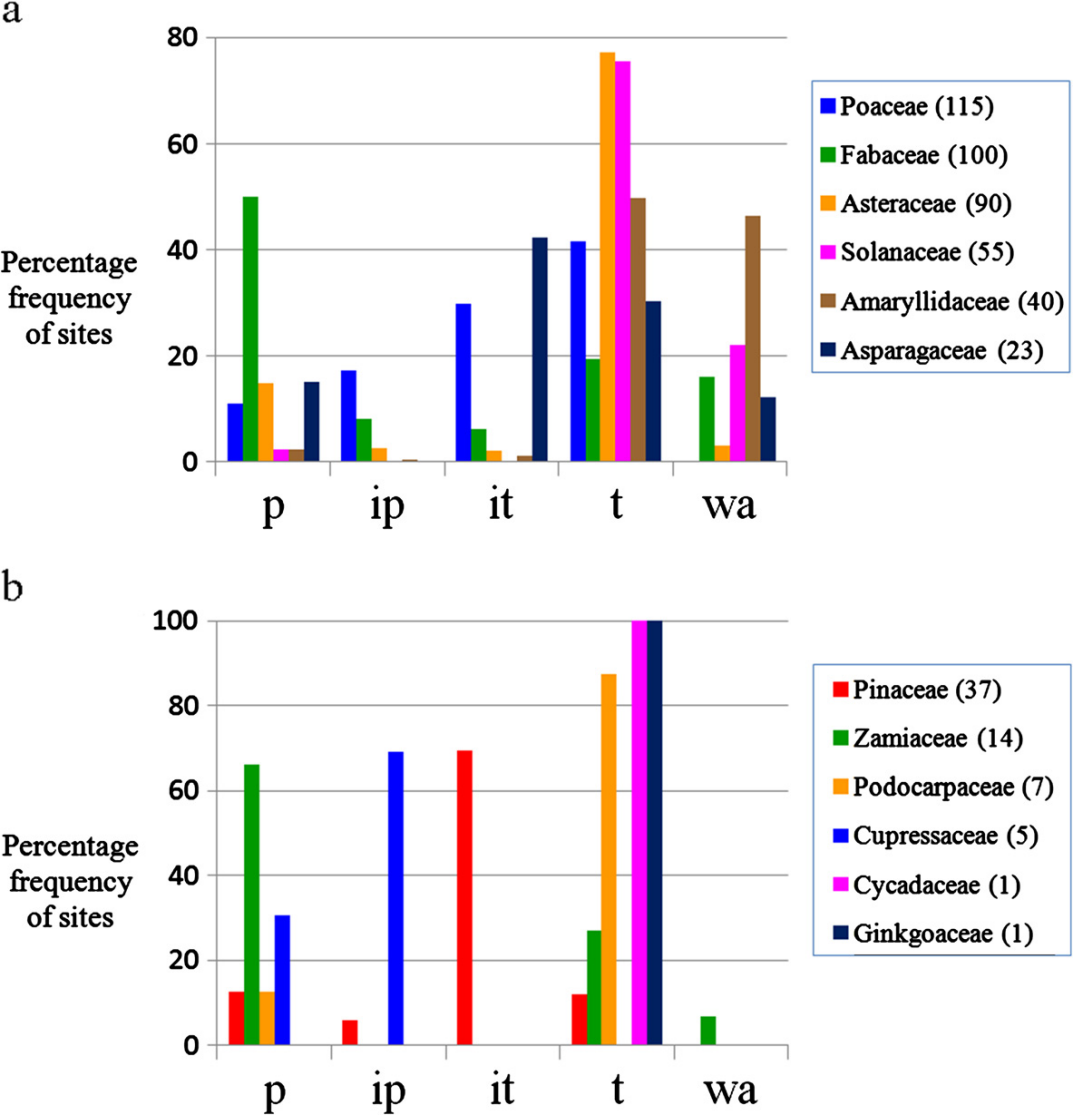
**Percentage frequency of rDNA sites along the chromosome arm in the most sampled families of angiosperms (a) and gymnosperms (b).** The sites were positioned in one of four equal-sized regions of the arm or spanning the whole arm. p, proximal; ip, interstitial-proximal; it, interstitial-terminal; t, terminal; wa, whole arm. The number of karyotypes analyzed in each family appears in brackets.

The distribution of rDNA sites may also be related to karyotype characteristics or chromosomal organization of specific taxa. For example, during the interphase, centromeres are often clustered in one pole of the cell and telomeres in the opposite pole, as a consequence of the anaphase movement. This kind of chromosome distribution in the nucleus, named Rabl orientation, seems to be more common in species with large chromosomes than in those with small ones [57,58]. In wheat, for example, with large chromosomes and large genome, telomeres are associated with the nuclear envelope in one pole of the nucleus whereas in *Arabidopsis* species, with much smaller chromosomes and genomes, telomeres are organized around nucleolus [57,59,60].

The distribution of sites by sub-regions of the chromosome arm (p, ip, it, t) is also not the same in different taxa. In Amaryllidaceae, Asteraceae, Cycadaceae, Ginkgoaceae, Poaceae, Podocarpaceae and Solanaceae the sites were predominantly terminal, while in Asparagaceae, Cupressaceae, Fabaceae, Pinaceae and Zamiaceae, the most common position was proximal to interstitial (Figure 5). Within some families, such as Solanaceae, the distribution of sites was similar in most genera (Additional file 2: Figure S3), while in others, such as Fabaceae, the sites were predominantly or exclusively proximal in some genera, such as *Arachis*[61-64] and *Lens*[65], and mainly terminal in others, as *Phaseolus*[66,67] and *Vicia*[68-71] (Additional file 2: Figure S3). The stability of sites in Solanaceae may be related to the karyotype stability of this group [72] although conservation in chromosome number and morphology is not enough to ensure stability of rDNA site position as observed in other taxa with apparently stable karyotype, as *Citrus*[73] and *Pinus*[74].

**Figure 6:**
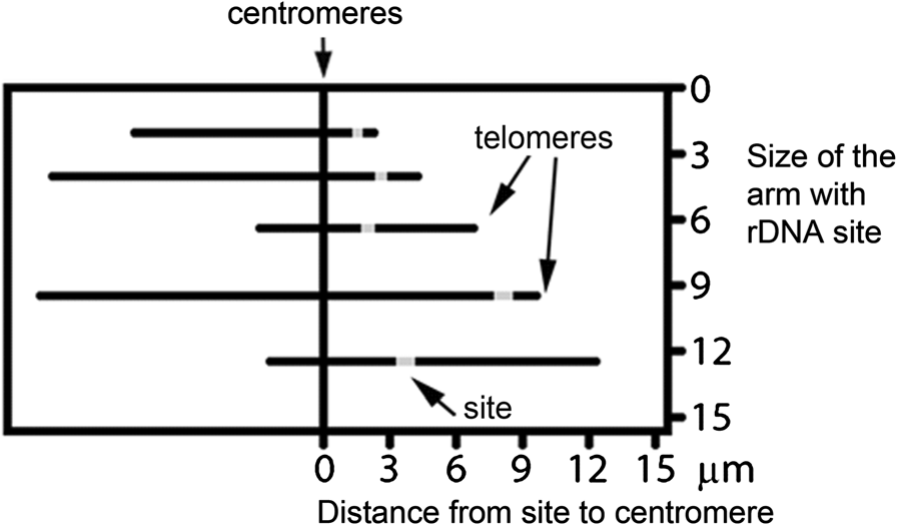
**Representation of chromosomes in the arm frame.** Chromosomes represented by lines were aligned in the vertical axis by centromeres. The arm with rDNA site (gray) was located at right side of the centromere, according to its size in Î¼m (based on Lima-de-Faria [9]).

### Distribution of rDNA sites in holokinetic chromosomes

A special case of rDNA distribution was observed in species with holokinetic chromosomes. This group is characterized by absence of a localized centromere (primary constriction) and the occurrence of a large kinetochore plate along almost its entire length [75]. Among 63 karyotypes with holokinetic chromosomes (42 species of 7 genera), the average number of sites per karyotype was 6.6 (6.6) and the mode and median were equal to 4.0, therefore, quite similar to those of angiosperms with monocentric chromosomes (Table 1). Although the number of sites was quite variable, 100% of them were located in the terminal region. In the genus *Rhynchospora*, for example, the number of sites ranged from 2 to ~30, but the position was always terminal [21,76]. Also in *Caenorhabditis elegans* and several species of insects with holokinetic chromosomes the 45S rDNA site is located terminally [77-79]. This suggests that in organisms with holokinetic chromosomes there are tighter constraints on the establishment of non-terminal sites. A possible explanation is that a secondary constriction in the interstitial region would interrupt the kinetochore plate along the holokinetic chromosome establishing a condition similar to a dicentric chromosome, with consequent errors in mitotic segregation [75]. However, this assumption has yet to be demonstrated and at least in Lepidoptera it does not seems to apply [80].

### Equilocality of rDNA in karyotypes with multiple sites

The equilocality or equal positioning of rDNA sites in relation to centromere or telomere has been previously observed in several genera [15,17,25,31,34]. To assess the frequency of equilocality of rDNA sites per chromosome complement, 243 diploid karyotypes with multiple sites were analyzed and the distance of each site to centromere and telomere were measured. Two sites were considered equilocated when they were at the same distance from the centromere or telomere, or at a distance less than 1/10 of the length of the largest chromosome arm bearing rDNA site. In relation to the centromere, 67.7% of karyotypes had at least two sites equilocalized and only 38.7% of karyotypes had all sites equally located. Regarding the telomere, the frequency of equilocality was significantly higher (89.9% and 73.8%, respectively - p<0.01).

Equilocality of rDNA sites seems to be promoted by ectopic recombination between regions of different chromosomes located in close proximity during prophase or interphase [39]. This spatial distribution could be facilitated by Rabl configuration in mitosis [15] or the bouquet configuration during meiotic prophase, where all telomeres attach to one pole of the nuclear envelope [81]. However, in species in which Rabl configuration does not occur, as Arabidopsis and human [82,83], as well as in those with holokinetic chromosomes [21,84], all rDNA sites are also terminal and equilocal, suggesting that Rabl orientation during interphase is not necessary to maintain the regularity in the position of rDNA. The bouquet configuration, on the other hand, is a more universal phenomenon [85] and could better explain the equilocality since it promotes the confluence of all telomeres to a limited region of the nuclear envelope. In this case, the dispersion of rDNA sites would occur mainly among chromosome regions that are equidistant from the telomere, while centromeres are not directly co-oriented in the bouquet (see also [42]). This may explain the high frequency of karyotypes with all sites equilocated in relation to telomeres (73.8%) and a relatively low frequency of equilocality in relation to centromeres (38.7%).

### Changes in the number of rDNA sites in relation to its position on chromosomes

During early meiotic prophase nuclei exhibit a highly polarized and clustered arrangement of chromosome ends, called ‘bouquet’. Assuming that the dispersion of sites is mediated mainly by the bouquet configuration, the variation in the number of rDNA sites should be higher when the sites are located in the terminal region, since only these regions are clustered by the bouquet. To evaluate the role of the site position on the variation in number of sites, a sample of 56 genera was analyzed, each one having species with single sites restricted to a sinlge position. Among 34 genera having terminal single sites, 20 (58.8%) presented also species with multiple sites and in 14 of them all sites were terminal. On the other hand, 11 genera had species with proximal single sites but only two out of these 11 genera (18.2%) had species with multiple sites and in both cases the sites were not proximal (Table 3). Therefore, terminal rDNA sites seem to have higher mobility than proximal ones.

**Table 3:** Distribution of multiple rDNA sites in genera having species with single sites restricted to only one chromosome region

		**Number of genera with multiple sites**		
**Position of single sites**	**Number of genera with single sites**^**a**^	**In any region**	**In the same region**	**p-value**^**b**^
Proximal	11	2	0	0.008
Interstitial	11	4	3	n.s.
Terminal	34	20	14	

^a^ Genera having species with single sites in one position and other species with single sites in a different position were excluded from this sample.

^b^ Comparisons with terminal region (Fisher’s exact test), n.s. non-significant.

The tight proximity of the telomeres in the bouquet and the highest rate of recombination in the terminal region seem to contribute to the higher rate of changes in the number rDNA sites on this region. Certainly, other chromosomal features should also influence the dispersion of these sites, leading to species with similar karyotypes and different distribution of sites. A clear example of divergent patterns in very similar karyotypes was observed in species of *Phaseolus*. Among 37 accessions of *P. vulgaris* analyzed there was a variation of six to 18 sites of 45S rDNA per diploid complement [32] while in 17 accessions of *P. lunatus* the number of sites was stable - only one pair of sites [66]. This extreme contrast in two species with very similar chromosome size and morphology, geographical distribution and cultivation history, suggests that other factors, such as association with mobile elements [86,87], may play a decisive role in site dispersion.

### Variability between diploids and polyploids

The variability between diploids and polyploids was analyzed at the intra-specific and intra-generic levels. In 19 out of 26 species (59 karyotypes) that had one or more polyploidy cytotypes, the number of sites per monoploid complement (SMC) was constant (Additional file 1: Table S2). In four of these 19 species there was variation in the number of sites per karyotype in different diploid cytotypes, but the tetraploid cytotype exhibited the same number found in one of the diploids. The position of the sites could be determined in only 18 out of 26 species. In 14 of them the position of the sites remained unchanged in diploids and polyploids (Additional file 1: Table S3).

To investigate the variability at the intrageneric level, 40 genera with two or more species were analyzed (species with intraspecific variability were not included). The number of SMC between different ploidy levels was stable in only 6 genera (15%). Among the remaining 34 genera the number of SMC in polyploidy species was lower than in diploids in 29 cases, and higher in only seven (Additional file 1: Table S4).

In general, polyploidy results in doubling of sites when it is of recent origin (intraspecific polyploidy), but when comparing different diploid and polyploid species of the same genus there was a clear trend to reduce the number of SMC in the latter. Considering that the period of time of evolutionary divergence is usually larger between species of the same genus than between cytotypes of the same species, it is reasonable to assume that older polyploids more often display "diploidized" numbers of rDNA sites, as observed in *Aristolochia*[88], *Nicotiana*[38], and *Avena*[89].

### The distribution of 45S rDNA sites in animals

The number and location of rDNA sites in other eukaryotes seem to follow some of the trends observed here, although we are not aware of such a similar compilation in other large groups of organisms. A quick look at the literature showed that the modal number of rDNA sites was 2 or 4 per cell in each one of the following samples: 49 species of Orthoptera [40], 31 Scarabaeinae bettles [90], 18 Lepidoptera [80], 38 triatomine (Heteroptera) [91], 13 representatives of five tribes of cichlid fishes [92], 61 rodents [42,93], 50 bats [93], and 56 species of reptiles [94]. The number of rDNA sites in this sample was quite variable within some groups, as rodents and grasshoppers, but much more conserved in others, as triatomine and bats.

The distribution of 45S rDNA sites per arm was distinct for different taxa. For example, in bats the sites were restricted to the short arm in all 7 families and 38 genera [93] whereas in reptiles they were largely concentrated on large arms or on microchromosomes [94]. The site distribution per chromosome arm region was more difficult to assess. However, most sites were located very close to the centromere of acrocentric chromosomes or at the terminal/subterminal region of the short or long arms, but only rarely they were found in the interstitial region. Curiously, in holokinetic chromosomes of Lepidoptera the rDNA sites were not restricted to the terminal position [80], as observed in plants. All together, these data suggest that the non-random distribution of rDNA sites observed in most taxa may be influenced by other important aspects of the chromosomal structure and organization, such as the amount of active mobile elements [87], nuclear DNA amount [26], and dynamics of interphase chromosomes [57].

## Conclusions

All these data together, indicate that: i, in angiosperms 45S rRNA genes occur preferentially on the short arms, concentrated on one or two clusters per haploid set, and in the terminal region of chromosomes; ii, in gymnosperms there are two different patterns: mainly interstitial sites in Pinaceae and mainly terminal or proximal sites in all other families; iii, the preference for the short arm is especially evident in very short arms of acrocentric chromosomes, where they often seem to span the whole arm; iiii, the location is more strictly controlled in holokinetic chromosomes where all 45S rDNA sites have been reported in the terminal position; iiiii, when the number of rDNA sites per karyotype increases, either by dispersion of repetitive units or by polyploidy, they tend to maintain the relative position of the original site. Although single rDNA loci are able to change its number and position in the genome, the establishment and dispersion of new sites appear to be constrained along the chromosome arms. Despite some general trends concerning the number and position of rDNA sites in plants, some species and even large taxonomic groups of plants deviate completely from these trends. Although there may be selection pressures favoring the terminal location, data of some groups indicate that there are no restrictions to the functioning of rDNA clusters in other positions or in higher number of sites and the forces driving the observed trends may be related to the chromosome architecture, nuclear organization and the dynamics of allelic and non-allelic recombinations.

## Methods

Information on the karyotype and the number and position of the 45S rDNA signals in mitotic metaphase cells of 253 original studies were used to build a database. The complete list of these articles and genera/families to which they refer is indicated in Additional file 1: Table S5. For the analysis of different aspects of the distribution of these sites, only part of the total sample was used since not all of the available karyotypes displayed all necessary information. Statistical analysis included Chi-square, Fisher’s exact test and proportion tests implemented in the software Epicalc (Brixton Health).

### Estimating the chromosomal morphology, size and the position of rDNA sites

The definition of metacentric, submetacentric and acrocentric chromosomes types was based on the arm ratio (ar) values, considering only metacentric chromosomes (ar < 1.5), submetacentric (ar ≥ 1.5 < 3.0) and acrocentric (ar ≥ 3.0, including those apparently telocentric [49]. The size in micrometers of the chromosomes bearing the rDNA sites was found in only 28 of 253 articles consulted. In other 194 articles there was a scale bar in micrometers, which allowed estimating the size of chromosomes by carefully measuring the size of the bar and the chromosomes. In 31 original works, where FISH data were presented without any information on chromosome size, this measurement was based on the size of the NOR bearing chromosome found in other publications for the same species. For example, for *Allium fistulosum* the number and relative position of rDNA sites were obtained from the work of Ricroch et al. [95] and the size of the chromosome bearing the NOR was based on measurements reported by Gernand et al. [96].

The position of the rDNA site was determined by two distinct approaches. First, based on metaphase photographs, the chromosome arm was divided into four regions of equal size [proximal (p), interstitial-proximal (ip), interstitial-terminal (it) and terminal (t)] and the site was positioned in one of them. When the rDNA signal seemed to coincide with the position of the centromere, it was referred to as "centromeric" (cen). Some sites appeared to span the entire chromosome arm, from the proximal to the terminal region, being named "whole-arm" sites (Additional file 1: Table S5). In the second approach, considering only idiograms, the absolute distance in micrometers from the site to the centromere was evaluated. In this case, the lengths from the centromere to the beginning of the site and from the end of the site to the telomere were estimated, as well as the size of the other chromosome arm. Whenever available, measurements provided in the original paper were used, otherwise they were estimated from the available idiograms or metaphases. All measurements were performed with Adobe Professional 9.0 (Adobe Inc.) in the digital version of the papers (Additional file 1: Table S6).

### Analysis of the distribution of rDNA sites by the arm frame method

The method proposed by Lima-de-Faria [7] was used aiming primarily to compare the distribution of rDNA sites with his data for secondary constrictions. Graphically, the chromosomes were represented by parallel lines, with centromeres aligned at the vertical axis and the rDNA bearing arm at the right side of this axis (Figure 6). The exact position of this arm in the graphic was given by its size, in both vertical and horizontal axes. Chromosomes with rDNA sites on both arms were represented twice in the graphic. In addition, the regions of higher and lower occurrence of these sites along the chromosome were identified by means of a graph of density of sites in the arm frame. Another analysis, similar to the arm frame, considered the size of the chromosome arm in micrometers in the Y axis and the relative distance of the site to the centromere in percentage on the X axis.

## Competing interests

The authors declare that they have no competing interests.

## Authors' contributions

FR acquired the data and drafted the manuscript. MG designed the study and revised the manuscript. All authors read and approved the final manuscript.

## Supplementary Material

Additional file 1 **Supplementary tables.** Tables showing details about karyotypes and analyses. (XLS 1198 kb) Click here for file

Additional file 2 **Supplementary figures.** Distribution of rDNA sites for the best sampled families and genera. (PDF 827 kb) Click here for file
