# Supplementary material for: Distribution of 45S rDNA sites in chromosomes of plants: Structural and evolutionary implications
Source: BMC Evol Biol. 2012 Nov 26;12:225. doi: 10.1186/1471-2148-12-225 (PMC3583730; doi:10.1186/1471-2148-12-225)

Figure S1. Relative distribution of rDNA sites along the chromosomal arm in families of angiosperms (a) and gymnosperms (b). The distance from the site to the centromere was represented as a percentage value in relation to the centromere-telomere length of the arm bearing the rDNA site. Number of karyotypes in parentheses.

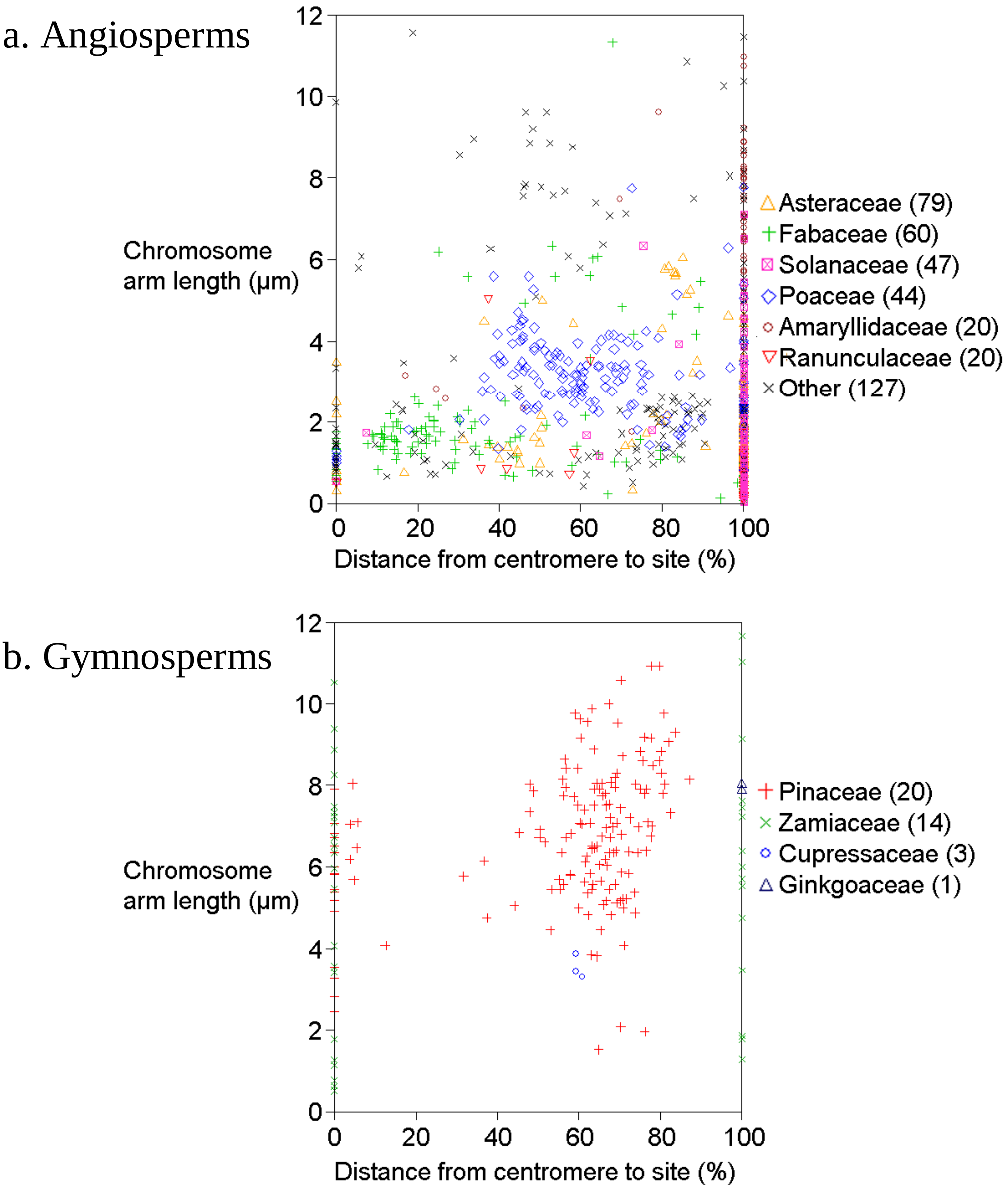

Figure S2. Distribution of rDNA sites in species of families Amaryllidaceae (a), Asteraceae (b), Fabaceae (c), Pinaceae (d), Poaceae (e), Ranunculaceae (f) and Solanaceae (g) considering the variation per genus. The diagonal limit represents the telomere of arms of different sizes. Number of karyotypes in parentheses.

a. Amaryllidaceae

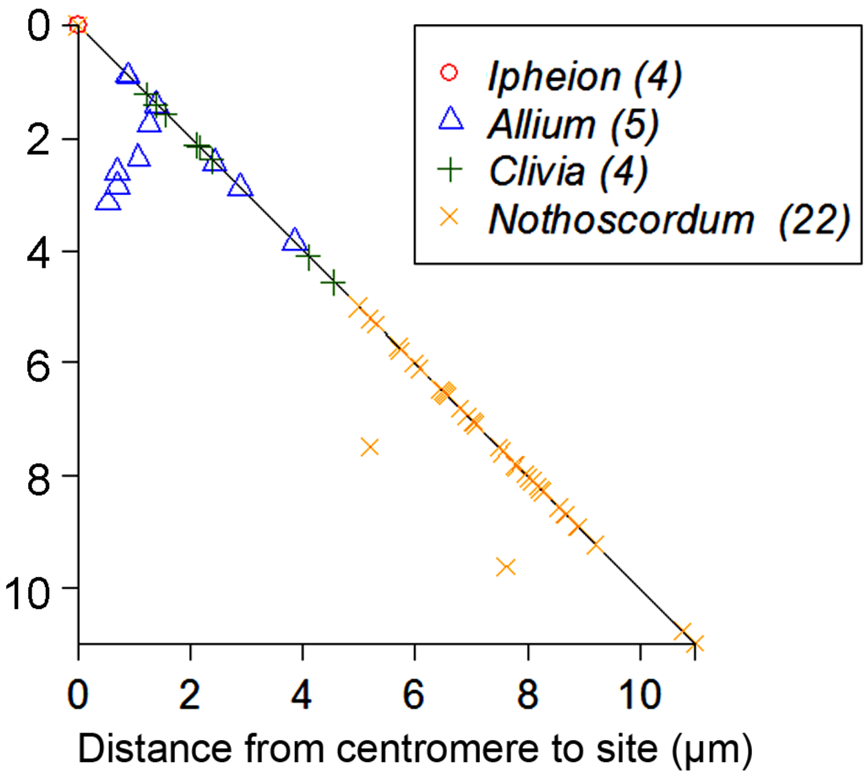

b. Asteraceae

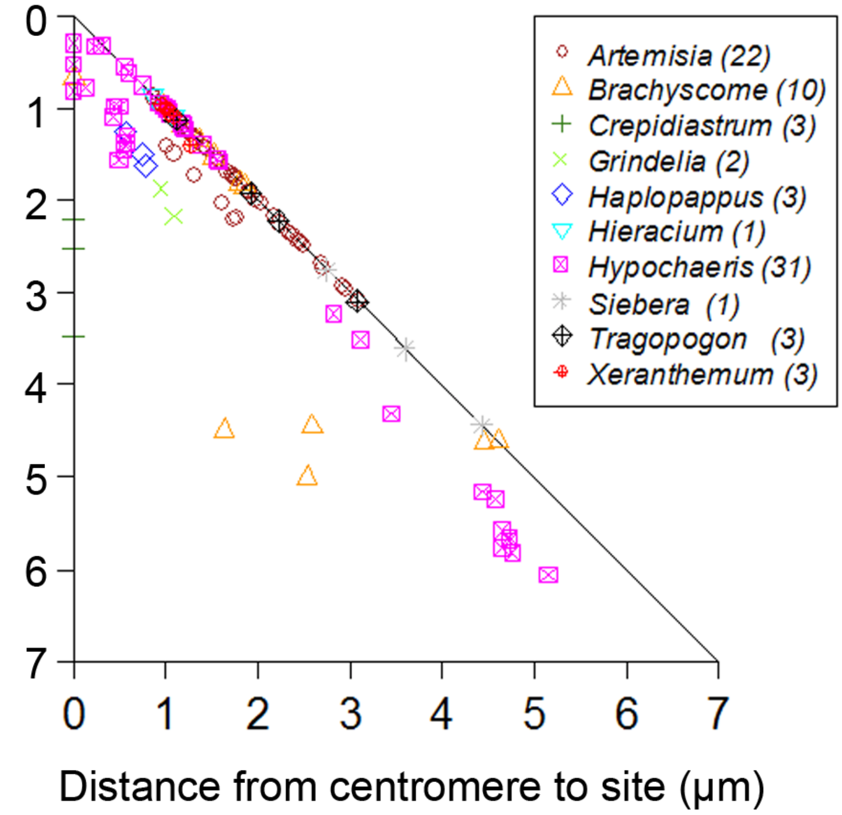

Figure S2.

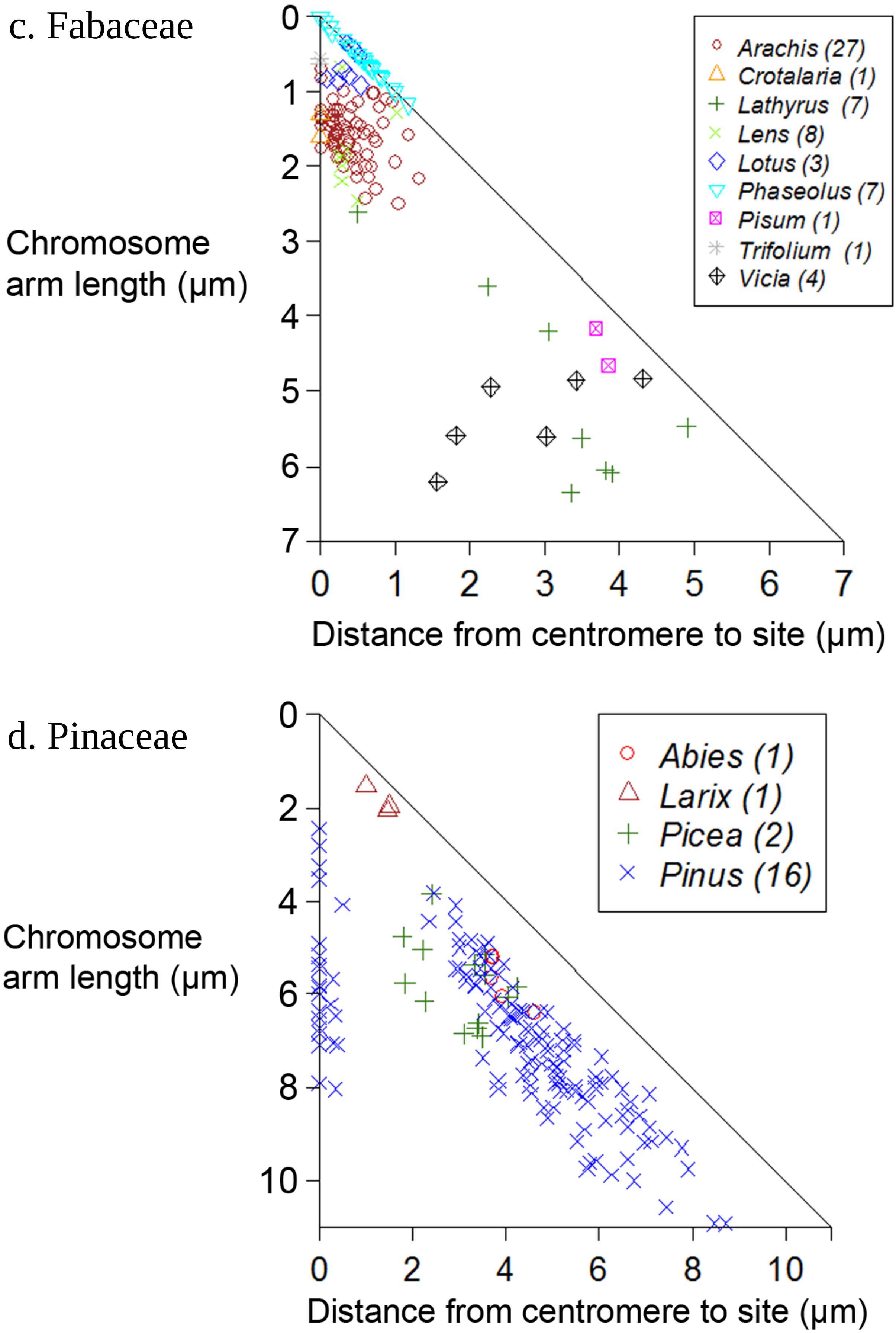
c. Fabaceae
Chromosome arm length (µm)
Distance from centromere to site (µm)
0
1
2
3
4
5
6
7
Arachis (27)
Crotalaria (1)
Lathyrus (7)
Lens (8)
Lotus (3)
Phaseolus (7)
Pisum (1)
Trifolium (1)
Vicia (4)
d. Pinaceae
Chromosome arm length (µm)
Distance from centromere to site (µm)
0
2
4
6
8
10
Abies (1)
Larix (1)
Picea (2)
Pinus (16)

Figure S2.

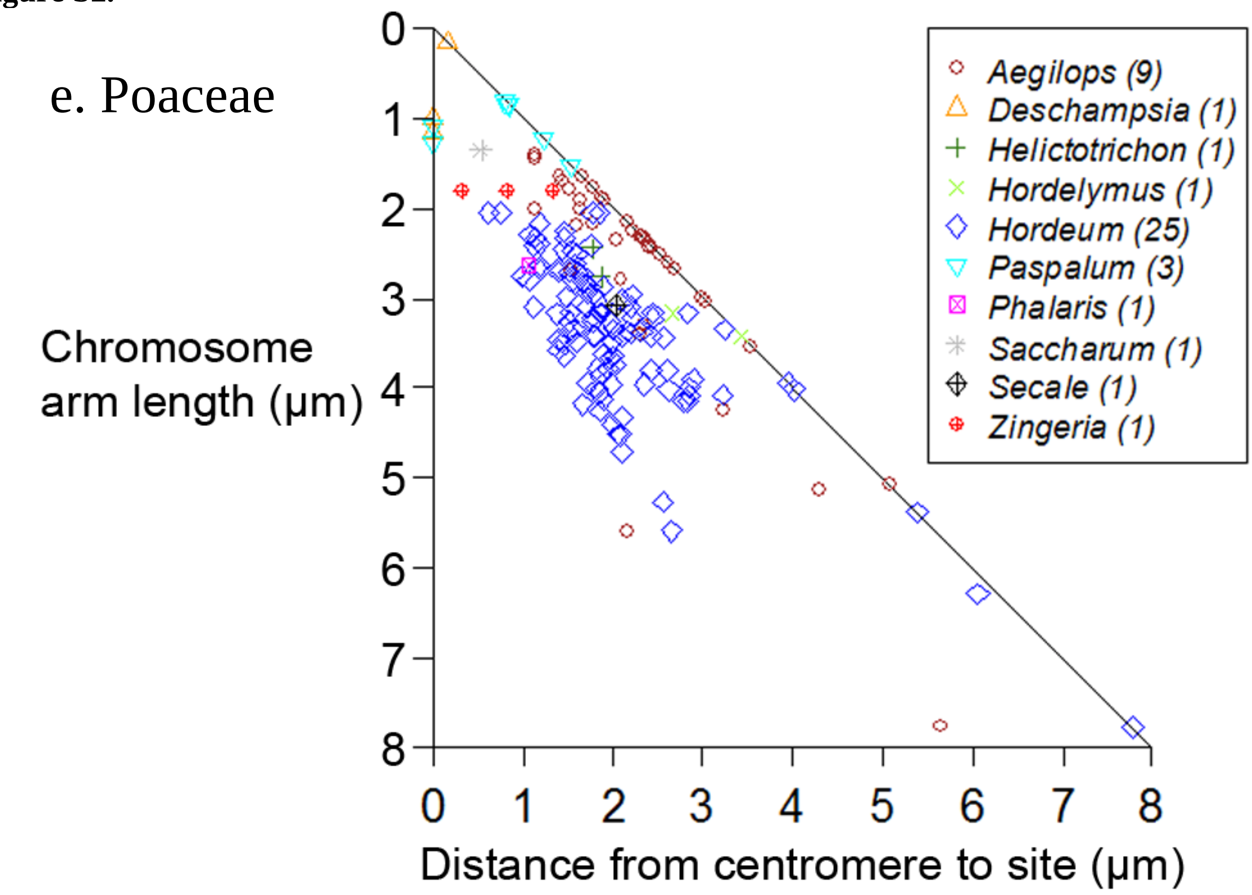

e. Poaceae
Chromosome arm length (µm)
Distance from centromere to site (µm)
Aegilops (9)
Deschampsia (1)
Helictotrichon (1)
Hordelymus (1)
Hordeum (25)
Paspalum (3)
Phalaris (1)
Saccharum (1)
Secale (1)
Zingeria (1)

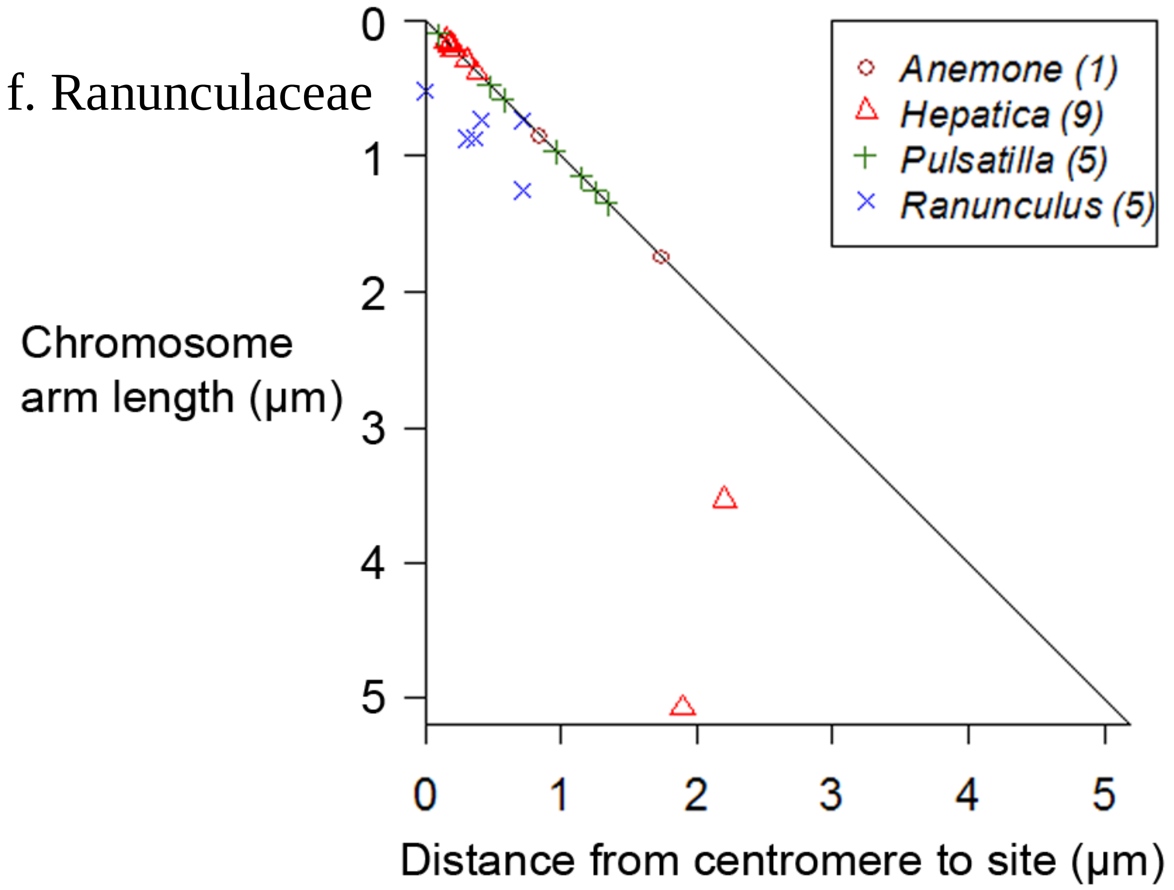

f. Ranunculaceae
Chromosome arm length (µm)
Distance from centromere to site (µm)
Anemone (1)
Hepatica (9)
Pulsatilla (5)
Ranunculus (5)

Figure S2.

g. Solanaceae

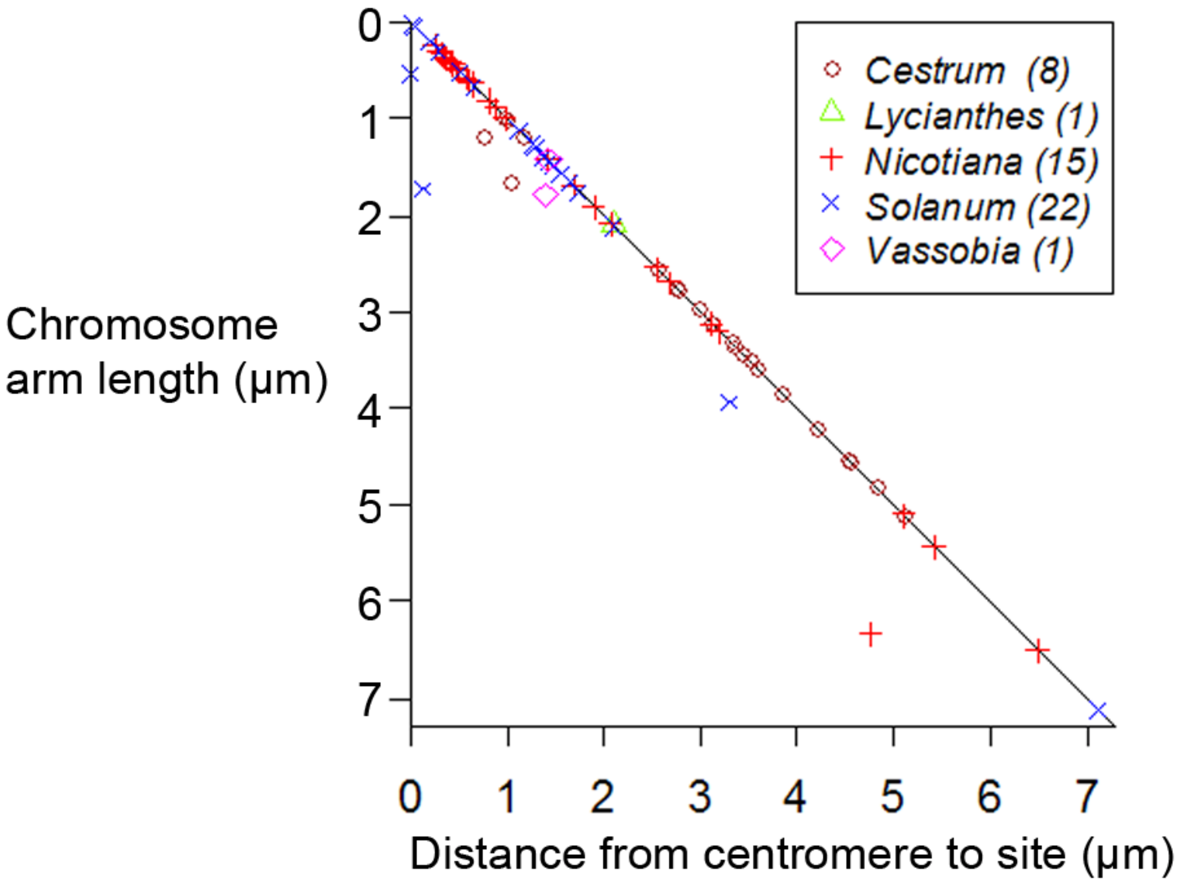

Figure S3. Percentage frequency of rDNA sites of the six most sampled genera of Fabaceae (a) and Solanaceae (b). Sites in the proximal (p), interstitial-proximal (ip), interstitial-terminal (it), and terminal (t) regions, as well as sites spanning the whole arm (wa), were indicated separately. Number of karyotypes in parentheses.

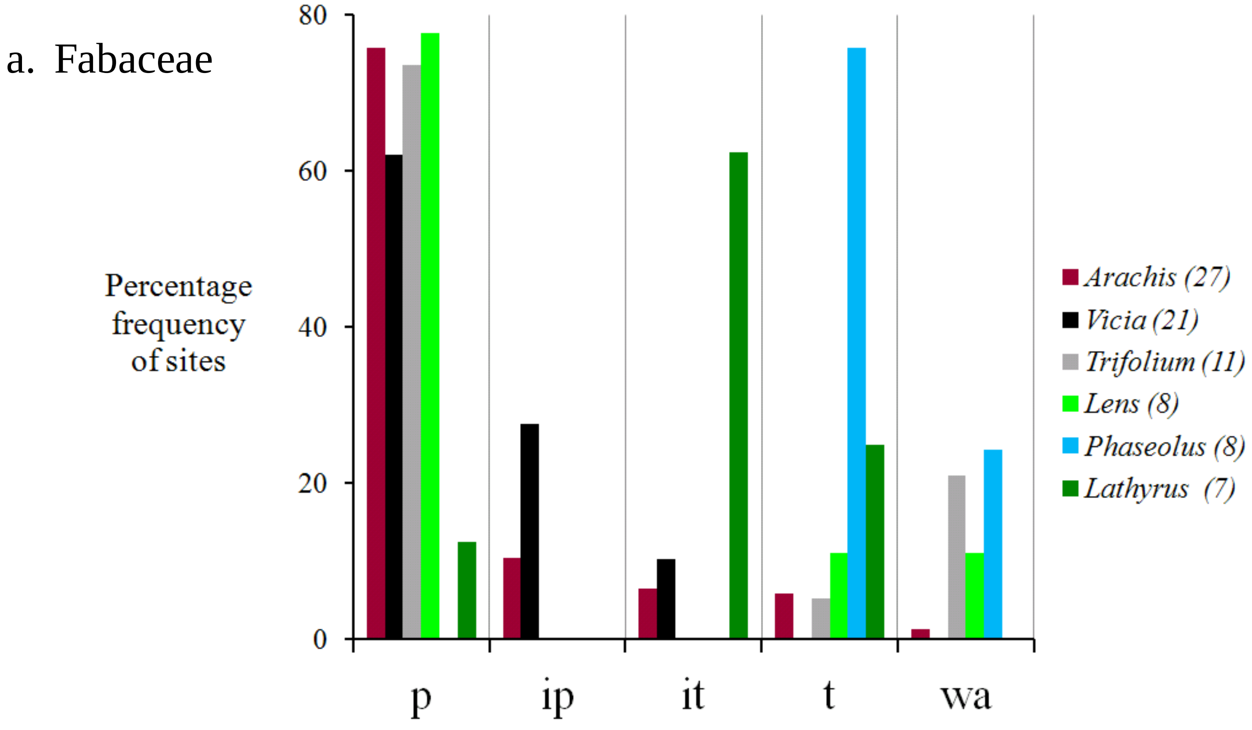

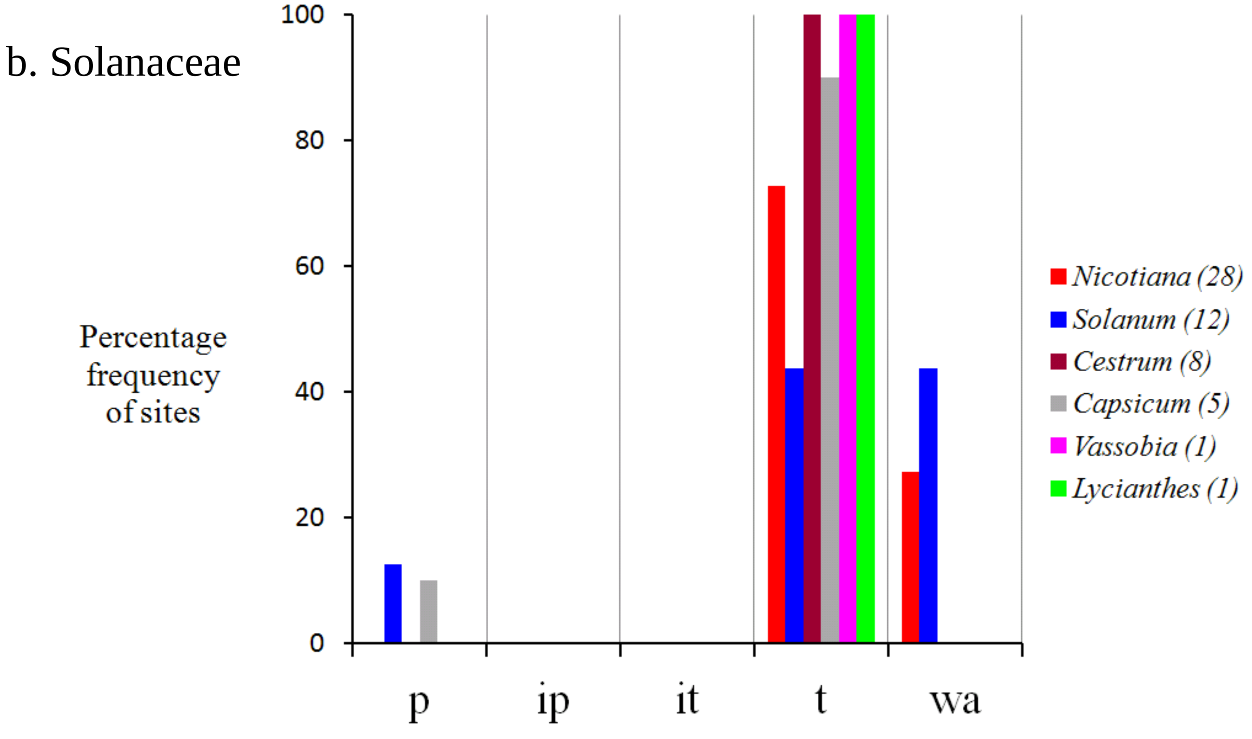

Supplement: Additional file 2 — Supplementary figures. Distribution of rDNA sites for the best sampled families and genera. (PDF 827 kb) [file 1471-2148-12-225-S2.pdf]
